# A PERCEIVED COMMUNITY TYPOLOGY IN OLDER KOREAN AMERICANS: IMPLICATIONS FOR MENTAL HEALTH

**DOI:** 10.1093/geroni/igae098.1567

**Published:** 2024-12-31

**Authors:** Nan Sook Park, Yuri Jang, Jeongsuk Kim, Jung Eun Ko, Soondool Chung, David Chiriboga

**Affiliations:** University of South Florida, Tampa, Florida, United States; University of Southern California, Los Angeles, California, United States; University of South Florida, Tampa, Florida, United States; Kyung Hee Cyber University, Seoul, Republic of Korea; Ewha Womans University, Seoul, Republic of Korea; University of South Florida, Tampa, Florida, United States

## Abstract

The health and well-being of older immigrants can be profoundly influenced by the neighborhoods in which they live. Considering that the physical and social characteristics of neighborhoods have the potential to either alleviate or exacerbate individuals’ mental health, the aims of this study were: (1) to identify perceived community typologies in older Korean Americans; and (2) to examine how these typologies are associated with feelings of loneliness and mental distress. We hypothesized that distinct community groups would be identified and that they would be differentially associated with mental health indicators as well as with background characteristics. Data were drawn from a survey with older Korean Americans aged 60 and older, collected during 2017−2018 in diverse locations (N = 2,138). To identify community typologies, a series of latent profile analysis (LPA) were conducted using 15 community-related variables in the three domains (neighborhood characteristics, social cohesion, ethnic attachment). After examining characteristics of the identified groups in relation to the study variables, hierarchical multiple regression models of loneliness and mental distress were estimated. Based on model evaluation criteria, an LPA model with five community groups was identified as the best fit. The five groups were identified as “safe/integrated” (10%), “safe/distant” (10%), “moderate integration” (38%), “marginal” (31%), and “vulnerable” (11%). Using the safe/integrated group as reference, the marginal and vulnerable groups were consistently associated with elevated feelings of loneliness and mental distress. The results suggest the need to understand profiles of community characteristics and their relationships with health/well-being among older immigrants.

